# Safety of sildenafil citrate: review of 67 double-blind placebo-controlled trials and the postmarketing safety database

**DOI:** 10.1111/j.1742-1241.2009.02254.x

**Published:** 2010-01

**Authors:** F Giuliano, G Jackson, F Montorsi, A Martin-Morales, P Raillard

**Affiliations:** 1 AP-HP, Neuro-Uro-Andrology, Department of Physical Medicine and Rehabilitation, Raymond Poincaré HospitalGarches, France; 2 Cardiac Department, St. Thomas’ HospitalLondon, UK; 3 Department of Urology, Università-Vita Salute San RaffaeleMilan, Italy; 4 Servicio de Urologia, Complejo, Hospitalario Carlos HayaMálaga, Spain; 5 Safety and Risk Management, Pfizer IncNew York, NY, USA

## Abstract

**Aim:**

To review special safety topics associated with sildenafil and to document the tolerability of 50- and 100-mg doses, overall and by age, in men with erectile dysfunction (ED).

**Methods:**

Data were collated from 67 double-blind placebo-controlled (DBPC) trials (> 14,000 men) conducted by the manufacturer and from the manufacturer’s postmarketing safety database (39,277 patients). The DBPC data were stratified by dose, starting dose and age (≥ 65 and ≥ 75 years). Special safety topics included cardiovascular risk, priapism, non-arteritic anterior ischaemic optic neuropathy (NAION), impaired renal and hepatic function, drug interactions (i.e. nitrates, cytochrome P3A4 inhibitors, other ED therapies and α-blockers) and incorrect use.

**Results:**

Sildenafil was well tolerated at a dose of 50 or 100 mg in men with ED, overall, in those aged ≥ 65 years, and in those aged ≥ 75 years. Analyses of the databases did not reveal any causal link between sildenafil and cardiovascular events, or any new safety risks relating to cardiovascular events, priapism, NAION, hearing loss or drug interactions. In the small number of men with moderate impairment of renal function or hepatic function who were treated with sildenafil in DBPC trials, the safety profile was similar to that in men with no impairment of renal or hepatic function. Overdose with sildenafil was rare in the ED population. No new safety issues, emerging trends or adverse reactions were identified in conjunction with overdose, dependence, abuse or misuse.

**Conclusion:**

This collated review confirms generally the good tolerability and established safety profile of sildenafil 50 and 100 mg in men with ED and reveals no new safety issues.

**Review Criteria**

Using a database of 67 double-blind placebo-controlled trials conducted by the manufacturer and the manufacturer’s postmarketing safety database, we reviewed special safety topics and, for the most frequently prescribed doses (50 and 100 mg), conducted a comprehensive assessment of the tolerability of sildenafil overall and by age (i.e. stratifying cut-offs of ≥ 65 and ≥ 75 years).

**Message for the Clinic**

Sildenafil 50 and 100 mg were generally well tolerated, including by elderly men. There was no causal link with cardiovascular events. Furthermore, no new safety risks relating to priapism, non-arteritic anterior ischaemic optic neuropathy or incorrect use were detected. In addition, there were no unexpected adverse reactions from drug interactions, and a similar safety profile was present in men with or without moderate impairment of renal or hepatic function.

## Introduction

Sildenafil citrate (VIAGRA®, Pfizer Limited, Sandwich, Kent, UK), approved by the United States Food and Drug Administration in March 1998 and by the European Medicines Agency in September 1998 for the on-demand treatment of erectile dysfunction (ED), was the first phosphodiesterase type 5 (PDE5) inhibitor licensed for this indication. Initial clinical trials of sildenafil were conducted in the United Kingdom (1), Europe (2) and the United States (3) and were followed by trials performed in Central and South America (4–6), Africa (7,8), Asia (9,10) and Australia (11). The effectiveness and safety of sildenafil for treating ED have been established in over 120 manufacturer-sponsored clinical trials with a cumulative exposure of more than 14,000 patient-years and in other independent studies (12,13). In most of the trials, sildenafil was administered on demand with a starting dose of 50 mg and subsequent adjustment to 25 mg or, more usually, 100 mg depending on toleration and efficacy, but it has also been studied in once-daily dosage (10, 25 and 50 mg) (14) and in single-doses up to 800 mg (15). Comprehensive reviews examining the efficacy and safety profile of sildenafil have been published (16,17). As of August 2008, sildenafil had been prescribed to more than 37 million men, and more than a billion tablets (averaging six per second) have been dispensed worldwide (18).

Treatment-related adverse events with PDE5 inhibitors such as sildenafil, vardenafil (Levitra®, Bayer AG, Leverkusen, Germany) and tadalafil (Cialis®, Eli Lilly Nederland B.V., Houten, the Netherlands) are generally mild to moderate, showing minor differences across the PDE5 inhibitor class (19,20). Headache, facial flushing, nasal congestion and dyspepsia are the most common adverse events (19,21–23). Postmarketing surveillance of PDE5 inhibitors has provided additional data that, in general, indicate a safety profile consistent with that reported in premarketing clinical studies (24–30). However, there are some gaps in the knowledge base. For example, the safety of PDE5 inhibitor therapy has not previously been reported by age,^1^ despite an increased prevalence of ED in older men (31). Given the ageing populations of the developed and developing world, with an expected increase in the prevalence of ED to more than 300 million men worldwide by 2025 (32), this will become increasingly important.

Using a database of all 67 of the manufacturer’s double-blind placebo-controlled (DBPC) trials and the manufacturer’s postmarketing safety database, we conducted a comprehensive assessment of the tolerability of sildenafil overall, by dose and by age (cut-offs of ≥ 65 and ≥ 75 years). The objective of this collated review was to assess special safety topics and to document the tolerability by age of the most frequently prescribed doses (50 and 100 mg).^2^

## Methods

### DBPC database

The DBPC database contains all routine safety data and serious adverse event data for the 67 DBPC sildenafil trials for the indication of ED, which were completed by June 1, 2007. Appropriate Institutional Review Board approval and patient informed consent were obtained for all trials.

Although sildenafil was studied in doses of up to 800 mg in phase I, single-dose, healthy volunteer studies (15), doses in the DBPC database ranged from 5 to 200 mg. However, most of the trials used the currently approved doses for the treatment of ED (25, 50 and 100 mg) and included a double-blind phase of 12 weeks (15). In 45 trials, the dose was flexible (most had dose adjustment at an interim visit according to efficacy and toleration while blinding was maintained), and in 22 trials, the dose was fixed. In most of the flexible-dose trials, the initial dose of sildenafil was 50 mg and most patients were titrated to 100 mg. In nearly all of the trials, sildenafil could be taken on demand approximately 1 h before sexual activity but not more than once daily. Many of the trials also included an open-label extension phase after the completion of the DBPC phase, in which most patients used sildenafil 100 mg.

Participants were generally required to have a documented history of ED of at least 3–6 months and to be in a stable sexual relationship. Trials that investigated conditions that contribute to the aetiology of ED generally required that the condition (e.g. diabetes mellitus) be stable or stable under treatment. Otherwise, the inclusion and exclusion criteria of the trials were broadly consistent with the Viagra prescribing information (15). In most of the trials, men receiving nitrate therapy and nitric oxide donors were excluded but, in some early trials, a few men randomised to sildenafil (*n* = 16) or placebo (*n* = 9) were taking glyceryl trinitrate ‘as required’. In some of the more recent trials, to avoid potential hypotension, any patient who was currently prescribed, taking, and/or likely to be treated with an α-blocker was either excluded from entering the trial or was not to take study medication within 4 h of dosing with the α-blocker.

There were no restrictions on other vasoactive and antihypertensive medications. Men with severe cardiac failure, unstable angina or a recent history (i.e. within 3–6 months) of stroke or myocardial infarction were excluded. Otherwise, efficacy and safety were assessed in men with a variety of comorbid conditions that share risk factors with ED, including diabetes mellitus, hypertension, cardiovascular disease (coronary artery disease, angina, myocardial infarction and stroke), radical prostatectomy, spinal cord injury and depression.

### Postmarketing safety database

From first launch in 1998 through September 2007 (when the database was closed), > 35 million patients are estimated to have used sildenafil worldwide for the treatment of ED. In accordance with Pfizer Pharmacovigilance Standard Operating Procedures, the postmarketing safety database contains all cases of adverse events that were reported spontaneously to Pfizer by healthcare professionals, persons other than healthcare professionals, national or local registries (when applicable) and health authorities, or that are systematically identified from the medical literature. It excludes cases from the sildenafil clinical trials programme (except for cases of serious adverse events). It also excludes cases representative of sildenafil indications other than ED (i.e. pulmonary hypertension). For cases reporting a dose, the dose represents the first daily dose taken by the patient at the onset of an adverse event.

### Analyses

In the clinical trials included in the DBPC database, the severity of each adverse event was categorised according to investigator judgment as mild (usually transient, required no special treatment and did not interfere with daily activities), moderate (low level of inconvenience and may have interfered with daily activities; usually ameliorated by simple therapeutic measures), or severe (interrupted daily activity and required systemic drug therapy or other medical treatment). A serious adverse event was defined as any untoward medical occurrence that resulted in death, was life threatening, required inpatient hospitalisation or prolongation of existing hospitalisation, or resulted in a persistent or significant disability/incapacity or a congenital anomaly/birth defect. Each event is presented using Medical Dictionary for Regulatory Activities (MedDRA) preferred terms (version 10.0) and with causality assessed by the trial investigator.

Using the DBPC database, incidence of adverse events, severe and serious adverse events and discontinuations were derived from the double-blind phase only. Data were analysed by dose (fixed-dose trials) and by modal flexible-dose (flexible-dose trials). Modal flexible-dose was defined as the dose the man was exposed to the longest during the trial period; if the exposure duration was equal for two different doses, the higher dose was selected as the modal dose. Data were also analysed by starting dosage (< 50, 50 and 100 mg) and by starting dose stratified by age groups. In this report, only the data for starting doses of 50 and 100 mg are considered. Death listings are reported for all phases of the study (e.g. including open-label extension).

Most postmarketing adverse event reports are submitted voluntarily, causality is not assessed, clinical information may be missing or incomplete, follow-up information may not be available, the magnitude of underreporting is unknown and many factors influence reporting (i.e. length of time since marketing, market share of the drug, publicity or media reports about the drug or an adverse event, the seriousness of an adverse reaction, regulatory actions and awareness by healthcare professionals and consumers of adverse event reporting and litigation) (33,34). Furthermore, because the source of medication is usually not captured, reports related to non-genuine products can enter a database. For example, an estimated 44% of tablets labelled ‘Viagra’ and sold over the Internet is counterfeit (35). Counterfeit ED medications may contain none or an unknown quantity of the purported active ingredient, other possibly active ingredients of unknown efficacy and safety, or even toxic ingredients (36). Therefore, the spontaneous reporting system yields reporting rates, not incidence rates, and it is generally not appropriate to make comparisons between drugs in reporting rates. The most important role of the spontaneous reporting system is for signal detection (34).

Common adverse events were defined as those occurring in > 2% of men in ≥ 1 treatment or age group in the DBPC database, or at a reporting rate ≥ 1% in at least one of the two dosage groups in the postmarketing safety database.

### Special safety topics

Several safety topics of special interest were selected for more detailed analysis, including certain adverse events [e.g. cardiovascular risk, priapism and non-arteritic anterior ischaemic optic neuropathy (NAION)] and events that might arise from intrinsic factors (e.g. impairment of renal and hepatic function), drug interactions [e.g. with nitrates, cytochrome P3A4 (CYP3A4) inhibitors, concomitant ED medications and α-blockers] or incorrect use (e.g. overdose and abuse). Some of these topics (cardiovascular risk and impaired renal and hepatic function) are medical history factors, which are not coded in the postmarketing safety database and therefore could not be searched in that database. Cardiovascular risk was assessed using the DBPC database, in men with cardiovascular disease, receiving antihypertensive medications, or with diabetes. Also using the DBPC database, baseline laboratory parameters were used to identify moderate renal impairment (defined as > 1.5 times the upper limit of normal blood urea nitrogen/urea and creatinine) and moderate hepatic impairment [defined as > 1.5 times the upper limit of normal for ≥ 2 tests, including aspartate aminotransferase (AST), alanine aminotransferase (ALT), alkaline phosphatase and total bilirubin]. MedDRA-preferred terms were used to search for priapism, NAION, hearing loss and incorrect use [although cases having the intent to self-harm (e.g. suicide or suicide attempt) were excluded from the overdose analysis]. Comprehensive lists of α-blockers, nitrates, nitric oxide donors, CYP3A4 inhibitors and concomitant ED medications (37) were used to search for drug interactions.

## Results and discussion

### Databases

The DBPC database contained more than 14,000 men, who were enrolled and received at least one dose of study drug. The mean age of sildenafil and placebo patients in the 54 parallel design trials was 55 years, with the majority aged ≥ 45 years; similar trends were observed for the 13 cross-over trials. Most of the men were white. At the time of enrolment ED had persisted, on average, for 4.5 years; it was most frequently organic in origin. The most common concomitant illnesses were of cardiovascular aetiology or associated with cardiovascular risk factors [i.e. hypertension, diabetes mellitus (non-insulin dependent), hyperlipidaemia, hypercholesterolaemia, coronary artery disease and a history of myocardial infarction] reflecting the age-related prevalence of these conditions. Benign prostatic hyperplasia and localised prostate cancer, treated with transurethral prostatectomy and radical prostatectomy, respectively, were also relatively common, as were conditions associated with an elderly or ageing population (e.g. osteoarthritis and visual disturbance).

Consistent with a population of advancing age and with the underlying comorbidity frequently seen in patients with ED, the most common medications taken were those used to treat hypertension, hyperlipidaemia, diabetes mellitus, arthritic conditions and skeletal muscular injuries, and to prevent myocardial infarction. Thus, the DBPC database includes a heterogeneous ED population that is representative of the ED population as a whole.

The postmarketing safety database identified 39,277 sildenafil patients with adverse events through July 15, 2007. The vast majority of these were reported spontaneously by health or non-health professionals (Table 1). The first daily dose was identified in slightly less than half of the cases (i.e. > 25 and ≤ 50 mg, *n* = 12,843; > 50 and ≤ 100 mg, *n* = 5066). Demographical and case characteristics, where known, were comparable to those observed for the DBPC database, in that the majority of the population was ≥ 50 years of age and the majority of cases reported a first total daily dose of 25–100 mg. Most of the cases were non-serious (80.4%) and approximately half (54%; 21,334/39,277) of the men reported a case outcome. Of the cases with known case outcomes, nearly half (49%; 10,498/21,334) reported an outcome of recovered/recovering/recovering with sequelae, and very few reported a fatal outcome (6.1%; 1303/21,334). Medical history is not a coded search function in the postmarketing safety database, but concomitant medications were consistent with common concomitant medications used by men in the DBPC database.

**Table 1:** Sources of cases populating the postmarketing safety database

Source	Number (%)
**Spontaneous**	
Health professional	15,894 (40.5)
Non-health professional	22,397 (57.0)
Total	38,391 (97.5)
**Health authority**	
Health professional	658 (1.7)
Non-health professional	21 (0.1)
Total	679 (1.7)
Published report	307 (0.8)*
Total	39,277 (100)

*The international literature is carefully screened daily to identify all relevant cases. Although an attempt is consistently made to avoid counting events twice, there may be a few duplicate cases, i.e. reported in observational studies published in the literature (e.g. the International Men’s Health Study (29) and Prescription Event Monitoring study (24)) and as spontaneous postmarketing cases.

### Tolerability of 50 mg vs. 100 mg

**Figure 1:**
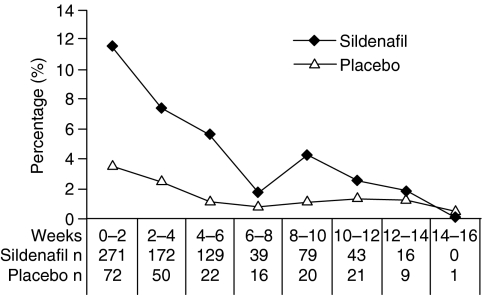
Rate of treatment-related adverse events over time collated from 17 randomised, double-blind, placebo-controlled, flexible-dose trials (sildenafil 25–100 mg, *n* = 2362; placebo, *n* = 1986). Treatment periods of up to 4 months were divided into 2-week intervals; the number of patients who experienced any adverse event was recorded for each interval and divided by the total number of patients who received treatment during that interval (38)

The safety profile of sildenafil, based on data from the DBPC database that included more than 7500 men treated with sildenafil 50 or 100 mg (Tables 2–4), remained consistent with that presented in the original regulatory submissions from approximately 10 years ago. Very rare were the serious adverse events of priapism (*n* = 11 patients), NAION (*n* = 0 patients) and hearing loss (*n* = 1 patient of unilateral loss considered to be embolic in aetiology), which are discussed in detail in subsequent sections of this review. Common adverse events in men treated with sildenafil were those related to the pharmacology of PDE5 inhibition, such as headache, vasodilation and facial flushing. These were reported in a higher incidence of men treated with sildenafil than with placebo (Table 4) and in a comparable incidence to that documented in the Viagra product information leaflet (15). Previously reported data collated from 17 of the randomised, DBPC, flexible-dose trials showed that the rate of these common adverse events decreased markedly over a 16-week treatment period such that, during the first 4–6 weeks of treatment, the rate among sildenafil-treated patients was higher than that for placebo-treated patients but, by 8 weeks and thereafter, the rate was similar between sildenafil- and placebo-treated patients (Figure 1) (38).

**Table 4:** Common treatment-related adverse events, stratified by dose and age, in 67 double-blind placebo-controlled trials

	Event by disorder system, number (%) of patients with event [with severe event]*								
	Eye			GI	Nervous system		Respiratory	Vascular	
Trial design dosage (*n*)† Age group (*n*)	Chromatopsia	Cyanopsia	Visual disturbance	Dyspepsia	Dizziness	Headache	Nasal congestion	Flushing	Hot flush
**Fixed-dose**									
Sildenafil 50 mg (804)	4 (0.5)	1 (0.1)	1 (0.1)	35 (4.4) [3]	14 (1.7) [1]	116 (14.4) [9]	15 (1.9) [1]	107 (13.3) [1]	27 (3.4) [1]
Sildenafil 100 mg (1373)	17 (1.2) [1]	19 (1.4)	35 (2.5)	64 (4.7) [6]	18 (1.3)	167 (12.2) [13]	29 (2.1) [1]	130 (9.5) [5]	30 (2.2)
Placebo (1623)	0	1 (0.1)	1 (0.1)	7 (0.4)	9 (0.6)	39 (2.4) [1]	2 (0.1)	16 (1.0)	3 (0.2)
**Flexible-dose**									
Sildenafil 50 mg (2060)	8 (0.4)	14 (0.7)	12 (0.6)	50 (2.4) [6]	58 (2.8)	264 (12.8) [21]	42 (2.0) [3]	205 (10.0) [2]	28 (1.4) [1]
Sildenafil 100 mg (3479)	13 (0.4)	36 (1.0)	23 (0.7)	101 (2.9) [5]	53 (1.5)	305 (8.8) [19]	65 (1.9) [3]	271 (7.8) [4]	12 (0.3)
Placebo (4979)	2 (0)	1 (0)	5 (0.1)	15 (0.3) [1]	40 (0.8) [2]	155 (3.1) [11]	12 (0.2)	65 (1.3) [1]	7 (0.1)
**All trials**‡									
**Sildenafil 50 mg, initial dose**									
All ages (6207)	24 (0.4)	51 (0.8)	35 (0.6)	181 (2.9)	127 (2.0)	705 (11.4)	125 (2.0)	577 (9.3)	56 (0.9)
< 65 years (5003)	21 (0.4)	42 (0.8)	27 (0.5)	146 (2.9)	103 (2.1)	587 (11.7)	107 (2.1)	472 (9.4)	47 (0.9)
≥ 65 years (1203)	3 (0.2)	9 (0.7)	8 (0.7)	35 (2.9)	24 (2.0)	118 (9.8)	18 (1.5)	105 (8.7)	9 (0.7)
≥ 75 years (134)	1 (0.7)	0	2 (1.5)	9 (6.7)	0	13 (9.7)	1 (0.7)	13 (9.7)	1 (0.7)
**Sildenafil 100 mg, initial dose**									
All ages (1337)	16 (1.2) [1]	19 (1.4)	35 (2.6)	64 (4.8) [6]	18 (1.3)	163 (12.2) [13]	28 (2.1) [1]	124 (9.3) [5]	23 (1.7)
< 65 years (1026)	10 (1.0) [1]	15 (1.5)	32 (3.1)	49 (4.8) [4]	12 (1.2)	134 (13.1) [10]	26 (2.5) [1]	98 (9.6) [5]	18 (1.8)
≥ 65 years (308)	6 (1.9)	4 (1.3)	3 (1.0)	15 (4.9) [2]	6 (1.9)	29 (9.4) [3]	2 (0.6)	26 (8.4)	5 (1.6)
≥ 75 years (37)§	1 (2.7)	0	1 (2.7)	3 (8.1)	1 (2.7)	2 (5.4)	0	3 (8.1)	1 (2.7)
**Placebo**									
All ages (6602)	2 (0)	2 (0)	6 (0.1)	22 (0.3) [1]	49 (0.7) [2]	194 (2.9) [12]	14 (0.2)	81 (1.2) [1]	10 (0.2)
< 65 years (5294)	2 (0)	2 (0)	3 (0.1)	21 (0.4) [1]	40 (0.8) [1]	161 (3.0) [12]	12 (0.2)	63 (1.2) [1]	9 (0.2)
≥ 65 years (1303)	0	0	3 (0.2)	1 (0.1)	9 (0.7) [1]	33 (2.5)	2 (0.2)	18 (1.4)	1 (0.1)
≥ 75 years (128)	0	0	0	0	0	2 (1.6)	0	1 (0.8)	0

AE, adverse event; DC, discontinuation; GI, gastrointestinal.

* Events occurring in > 2% of men in ≥ 1 group. An event was categorised according to investigator judgment as severe if it interrupted daily activity and required systemic drug therapy or other medical treatment. Listed are DCs, dose reductions and temporary DCs that were caused by an AE. Severe events are shown only when ≥ 1 occurred. Because of the small number of severe events, percentages are not given.

† In flexible-dose trials, dose is the modal dose, the dose that the patient was exposed to the longest during the study period. If the duration was the same for two different doses, the higher dose was selected as the modal dose of the patient.

‡ Sum of patient numbers in the ‘< 65 years’ group plus the ‘≥ 65 years group’. do not total the numbers in the ‘All ages’ group because age was missing for one patient (50 mg) and three patients (100 mg).

§ In addition to the tabulated events, there were also 1 (2.4%) case each of iris disorder (mild), abdominal discomfort (mild), diarrhoea haemorrhagic (moderate), gastroesophageal reflux (mild), nausea (mild), fatigue (moderate), heart rate increase (mild), musculoskeletal pain (moderate), pain in extremity (moderate) and hypoesthenia (mild).

**Table 2:** Overview of adverse events and discontinuations stratified by dose, in 67 double-blind placebo-controlled trials

	Fixed-dose trials			Flexible-dose trials		
	Sildenafil dose			Modal sildenafil dose*		
Adverse events†	50 mg (*N* = 804)	100 mg (*N* = 1373)	Placebo (*N* = 1623)	50 mg (*N* = 2060)	100 mg (*N* = 3479)	Placebo (*N* = 4979)
**AE, number of events**						
All causality	1101	1419	769	1807	2768	2491
Treatment-related	501	781	181	1077	1421	668
**AE, number (%) of patients**						
All causality	498 (61.9)	655 (47.7)	489 (30.1)	951 (46.2)	1549 (44.5)	1572 (31.6)
Severe	51 (6.3)	56 (4.1)	41 (2.5)	87 (4.2)	102 (2.9)	127 (2.6)
Serious	17 (2.1)	19 (1.4)	23 (1.4)	37 (1.8)	55 (2.6)	67 (1.3)
DC	14 (1.7)	20 (1.5)	19 (1.2)	50 (2.4)	27 (0.8)	49 (1.0)
Dose reduction or temporary DC	20 (2.5)	18 (1.3)	17 (1.0)	118 (5.7)	59 (1.7)	68 (1.4)
Treatment-related	295 (36.7)	436 (31.8)	136 (8.4)	655 (32.3)	885 (25.4)	480 (9.6)
Severe	21 (2.6)	19 (1.4)	6 (0.4)	35 (1.7)	29 (0.8)	19 (0.4)
Serious	1 (0.1)	1 (0.1)	1 (0.1)	3 (0.1)	5 (0.1)	3 (0.1)
DC	8 (1.0)	10 (0.7)	7 (0.4)	28 (1.4)	10 (0.3)	18 (0.4)
Dose reductions or temporary DC	1 (0.1)	8 (0.6)	1 (0.1)	102 (5.0)	29 (0.8)	32 (0.6)

AE, adverse event; DC, discontinuation.

* Modal dose: dose the patient was exposed to the longest during the study period. If the duration was the same for two different doses, the higher dose was selected as the modal dose of the patient.

† An event was categorised according to investigator judgment as severe if it interrupted daily activity and required systemic drug therapy or other medical treatment. A serious adverse event was defined as any untoward medical occurrence that resulted in death, was life threatening, required inpatient hospitalisation or prolongation of existing hospitalisation, or resulted in a persistent or significant disability/incapacity or a congenital anomaly/birth defect. Listed are DCs, dose reductions and temporary DCs that were caused by an AE.

The safety profiles of sildenafil 50 and 100 mg in the DBPC trials were comparable, in that there was no apparent increase in the incidence of men with all-causality or treatment-related adverse events among those receiving 100 mg as a fixed or flexible-dose compared with those receiving 50 mg as a fixed or flexible-dose (Tables 2 and 4). The exception to this was a known increased incidence of men with transient altered colour vision (chromatopsia and cyanopsia) at doses of ≥ 100 mg (15). Indeed, for some adverse events, the incidence was higher among those receiving 50 mg than among those receiving 100 mg. For example, headache [fixed-dose: 14.4% (50 mg) vs. 12.2% (100 mg); modal flexible-dose: 12.8% (50 mg) vs. 8.8% (100 mg)] and flushing [fixed-dose: 13.3% (50 mg) vs. 9.5% (100 mg); flexible-dose: 10.0% (50 mg) vs. 7.8% (100 mg)] (Table 4). It is likely that the differences between fixed-doses are clinically meaningless and that those between flexible-doses reflect enrichment of the 50-mg group with patients having tolerability issues that prevented dosage increase, such that their most frequent dose throughout the study was 50 mg.

In the population as a whole and in all subgroups stratified by age, the overall incidence of men with adverse events was similar in those initiating treatment with 100 mg vs. 50 mg, except for a slight dose-related increase in the incidence of treatment-related adverse events among older men (Table 3). Among the most common treatment-related adverse events, the incidence was generally similar with a starting dose of 100 mg vs. 50 mg (Table 4). The exception to this was headache in men aged ≥ 75 years, which occurred in 5.4% (2/37) of those starting with 100 mg and in 9.7% (13/134) of those starting with 50 mg. For most events, the incidence was also similar across age subgroups for each starting dose. Noteworthy exceptions to this were a higher incidence of men with dyspepsia in the subgroup of men aged ≥ 75 years, which was relatively small (50 mg, *n* = 134; 100 mg, *n* = 37), and a lower incidence of men with headache and nasal congestion in the subgroups of men aged ≥ 75 or ≥ 65 years.

**Table 3:** Overview of adverse events and discontinuations stratified by starting dose and age, in 67 double-blind placebo-controlled trials

	Overall*			< 65 years			≥ 65 years			≥75 years		
	50 mg	100 mg	PL	50 mg	100 mg	PL	50 mg	100 mg	PL	50 mg	100 mg	PL
Adverse events†	(*n* = 6207)	(*n* = 1337)	(*n* = 6602)	(*n* = 5003)	(*n* = 1026)	(*n* = 5294)	(*n* = 1203)	(*n* = 308)	(*n* = 1303)	(*n* = 134)	(*n* = 37)	(*n* = 128)
**AE, number of events**												
All causality	5539	1391	3260	4395	1038	2513	1144	353	747	123	44	75
Treatment-related	3011	758	849	2476	607	692	535	151	157	61	21	10
**AE, number (%) of patients**												
All causality	2935 (47.3)	638 (47.7)	2061 (31.2)	2344 (46.9)	487 (47.5)	1610 (30.4)	591 (49.1)	151 (49.0)	451 (34.6)	67 (50.0)	19 (51.4)	42 (32.8)
Severe	229 (3.7)	56 (4.2)	168 (2.5)	187 (3.7)	41 (4.0)	122 (2.3)	42 (3.5)	15 (4.9)	46 (3.5)	2 (1.5)	0	6 (4.7)
Serious	106 (1.7)	19 (1.4)	90 (1.4)	78 (1.6)	14 (1.4)	60 (1.1)	28 (2.3)	5 (1.6)	30 (2.3)	4 (3.0)	1 (2.7)	5 (3.9)
DC	113 (1.8)	25 (1.9)	89 (1.3)	88 (1.8)	16 (1.6)	64 (1.2)	25 (2.1)	9 (2.9)	25 (1.9)	5 (3.7)	2 (5.4)	3 (2.3)
Dose reduction or temporary DC	237 (3.8)	18 (1.3)	85 (1.3)	194 (3.9)	12 (1.2)	66 (1.2)	43 (3.6)	6 (1.9)	19 (1.5)	4 (3.0)	2 (5.4)	2 (1.6)
Treatment-related	1840 (29.6)	422 (31.6)	616 (9.3)	1510 (30.2)	329 (32.1)	496 (9.4)	330 (27.4)	93 (30.2)	120 (9.2)	40 (29.9)	13 (35.1)	7 (5.5)
Severe	81 (1.3)	19 (1.4)	25 (0.4)	68 (1.4)	14 (1.4)	23 (0.4)	13 (1.1)	5 (1.6)	2 (0.2)	0	0	0
Serious	9 (0.1)	1 (0.1)	4 (0.1)	6 (0.1)	1 (0.1)	3 (0.1)	3 (0.2)	0	1 (0.1)	0	0	0
DC	54 (0.9)	12 (0.9)	29 (0.4)	40 (0.8)	9 (0.9)	22 (0.4)	14 (1.2)	3 (1.0)	7 (0.5)	1 (0.7)	1 (2.7)	1 (0.8)
Dose reduction or temporary DC	176 (2.8)	8 (0.6)	34 (0.5)	150 (3.0)	6 (0.6)	28 (0.5)	26 (2.2)	2 (0.6)	6 (0.5)	3 (2.2)	1 (2.7)	0

AE, adverse event; DC, discontinuation; NA, data not available; PL, placebo.

* Sum of patient numbers in the ‘< 65 years’ group plus the ‘≥ 65 years group’. do not total the numbers in the ‘All ages’ group because age was missing for one patient (50 mg) and three patients (100 mg).

† An event was categorised according to investigator judgment as severe if it interrupted daily activity and required systemic drug therapy or other medical treatment. A serious adverse event was defined as any untoward medical occurrence that resulted in death, was life threatening, required inpatient hospitalisation or prolongation of existing hospitalisation, or resulted in a persistent or significant disability/incapacity or a congenital anomaly/birth defect. Listed are DCs, dose reductions and temporary DCs that were caused by an AE.

The majority of adverse events included in the postmarketing safety database were known sildenafil adverse drug reactions, similar to those reported in DBPC trials. Overall, the onset of more of the adverse events occurred in association with a total daily dose of 50 mg [i.e. > 25–50 mg; 32.7% (12,843/39,277)] than with a total daily dose of 100 mg [i.e. > 50–100 mg; 12.9% (5066/39,277)]. The higher number of reports in association with a total daily dose of 50 mg likely reflects that most adverse event reports are generated in men newly on treatment and that 50 mg is the recommended sildenafil staring dose. For most types of events, the reporting rates were similar between the 50- and 100-mg dose (Table 5). Not listed in Table 5 are reports of drug ineffective [50 mg, *n* = 3803 (29.6%); 100 mg, *n* = 2038 (40.2%)], drug effect decreased [50 mg, *n* = 1090 (8.5%); 100 mg, *n* = 397 (7.8%)], or ED [50 mg, *n* = 1132 (8.8%); 100 mg, *n* = 449 (8.9%)] because the efficacy of sildenafil is well established (12,13), an increase in dose or second-line treatment should be considered in cases in which sildenafil is ineffective (39), and it is the role of the healthcare professional to establish realistic treatment goals and expectations. Most men in whom sildenafil treatment failed responded successfully after re-education and counselling, which included information on patient and partner expectations, how to properly take the drug, titration to maximum dose and a minimum trial of eight attempts (40).

**Table 5:** In the postmarketing safety database, reporting rate of common adverse drug reactions for which the first total daily dose was 50 or 100 mg

	First total daily dose	
Disorder system Event, *n* (%)*	50 mg *N* = 12,843 reports†	100 mg *N* = 5,066 reports†
**Cardiac**		
Myocardial infarction	273 (2.1)	95 (1.9)
Palpitation	236 (1.8)	48 (0.9)
Tachycardia	165 (1.3)	36 (0.7)
**Eye disorders**		
Cyanopsia	233 (1.8)	189 (3.7)
Vision blurred	244 (1.9)	89 (1.8)
Visual disturbance	149 (1.2)	74 (1.5)
Gastrointestinal		
Dyspepsia	415 (3.2)	174 (3.4)
Nausea	276 (2.2)	82 (1.6)
**General and administration site**		
Chest pain	220 (1.7)	59 (1.2)
Drug interaction	248 (1.9)	121 (2.4)
Feeling hot	202 (1.6)	47 (0.9)
Malaise	130 (1.0)	40 (0.8)
**Injury, poisoning and procedural complications**		
Intentional drug misuse	85 (0.7)	66 (1.3)
Intentional overdose	153 (1.2)	121 (2.4)
Overdose	154 (1.2)	65 (1.3)
**Nervous system**		
Dizziness	502 (3.9)	167 (3.3)
Headache	1929 (15.0)	574 (11.3)
**Reproductive system**		
Priapism and related events‡	305 (2.4)	132 (2.6)
**Respiratory**		
Dyspnoea	163 (1.3)	54 (1.1)
Nasal congestion	530 (4.1)	156 (3.1)
**Skin and subcutaneous tissue**		
Erythema	304 (2.4)	59 (1.2)
**Vascular**		
Flushing	1367 (10.6)	409 (8.1)
Hot flush	183 (1.4)	26 (0.5)

* Events constituting ≥ 1% of reported events in ≥ 1 of the two dosage groups are listed.

† 50 mg = > 25–50 mg; 100 mg = > 50–100 mg.

‡ Priapism and erection increased.

In the DBPC database, the safety profiles of sildenafil 50 and 100 mg were comparable in that there were no apparent dose-related increases in the incidence of men with severe adverse events, serious adverse events, discontinuations caused by adverse events or dose reductions caused by adverse events in the population as a whole (Table 3) and in either the fixed-dose trials or the flexible-dose trials (Table 2). Across subgroups stratified by starting dose and age, there was a low incidence of men with treatment-related serious events (< 1%), severe events (< 2%), discontinuations caused by adverse events (< 3%) and dose reductions or temporary discontinuations caused by adverse events (≤ 3%) (Table 3).

The overall frequency of death was low in the DBPC database and was comparable between men using sildenafil (13/8691, 0.15%) and placebo (7/6602, 0.11%). Most deaths were in men aged ≥ 50 years. Among the deaths were six men using 50 mg (mean age, 56.8 years), of whom two were ≥ 65 years and one was ≥ 75 years; five men using 100 mg (mean age, 62.4 years), of whom three were ≥ 65 years and one was ≥ 75 years; and seven men using placebo (mean age, 67.2 years), of whom six were ≥ 65 years and one was ≥ 75 years. None of the deaths was considered to be treatment related.

In the postmarketing safety database, approximately 20% (7683/39,277) of patients were considered serious and 3.3% of patients (1310/39,277) had an outcome of death. Of the 882 deaths for which age was reported, the majority of cases [79.4% (700/882)] involved patients aged > 50 years. For serious adverse events and deaths, the order of magnitude differences between the incidence rates of the DBPC database and the reporting rates of the postmarketing safety database reflects the vastly different natures of these metrics, as described in Methods section.

### Cardiovascular risk

The prevalence of ED is increased in men at risk for cardiovascular disease (41–47). Further evidence for the relationship between ED and cardiovascular disease is that the most common organic cause of ED is vascular disease (31,48) and some of the most common comorbid diagnoses in men with ED are cardiovascular disease risk factors (i.e. hypertension, hyperlipidaemia and diabetes mellitus) (49). However, previous extensive study revealed no evidence that sildenafil use was associated with increased risk of adverse cardiovascular disease events (27,29,50–52).

Not surprisingly, given the known association between ED and cardiovascular disease, and between age and cardiovascular disease, the most commonly reported adverse events that resulted in death in the DBPC database were cardiac related (i.e. myocardial infarction, cardiac arrest, cardiac failure and coronary artery disease), and most of these were in men aged ≥ 50 years. However, it should be noted that the deaths were not attributed to sildenafil and that the overall cardiovascular death rate was slightly higher in the placebo groups (3 of 7 deaths) than in the sildenafil groups (4 of 11 deaths). Similarly, the incidence of men with serious cardiovascular events was comparable and not significantly different in the sildenafil and placebo groups [i.e. acute myocardial infarction (4.1% vs. 4.5%), chest pain (3.0% vs. 2.3%), coronary artery disease (2.7% vs. 5.3%), myocardial infarction (2.5% vs. 3.8%) and cerebrovascular accident (2.5% vs. 2.3%)], and none of these serious cardiovascular events was related to sildenafil treatment.

Based on available safety data, there is no evidence of a causal link between sildenafil and cardiovascular events. Safety data from the original regulatory submissions, postmarketing observational studies (24,29) and published literature demonstrate that sildenafil does not increase the rate of myocardial infarction or other serious cardiovascular events in men with ED. Furthermore, the safety profile of sildenafil in men with ED and diabetes mellitus (11,53,54), arterial hypertension (55) or cardiovascular conditions (56,57) is similar to that in men with ED without these conditions. The safety of sildenafil has not been studied in men with hypotension (blood pressure < 90/50 mmHg) or recent history of stroke or myocardial infarction, and its use is therefore currently contraindicated in men with these conditions (15).

### Priapism

Priapism is a rare adverse drug reaction with sildenafil. In the DBPC database, the incidence of men with priapism or related events was 0.1% (11/8691) in sildenafil recipients and < 0.1% (2/6602) in placebo recipients. Most cases reported events that were considered mild in severity; none that was considered as severe or defined as serious; and most of which resolved with no action, intervention or reduction of dose. Eight of the cases were associated with sildenafil 50 mg, including two that were characterised by multiple events and two (one of which resolved with a dose reduction) that were characterised by a duration of > 1 day. Three of the cases were associated with sildenafil 100 mg, including two that were characterised by multiple events that resolved, and two that were characterised by a duration of > 1 day and resolved with no action.

The postmarketing safety database was searched for priapism-related events (coded in the MedDRA as ‘priapism’ and ‘erection increased’). Comparison of priapism-related events as reported by healthcare professionals and consumers, respectively, shows that, although the same pathological entity gets reported (in most cases as a serious event), reports of healthcare professionals tend to use the term ‘priapism,’ whereas those of consumers tend to use the term ‘erection increased.’ The reporting rate was 2.5% for sildenafil 50 mg (320/12,843 total patients) and 2.7% for sildenafil 100 mg (138/5066 total patients). In contrast to the DBPC database, for which none of the cases of priapism was considered by the investigator to be severe or fulfilled the definition for ‘serious’, 56% (180/320) of the reports in the postmarketing safety database that were associated with sildenafil 50 mg and 64% (89/138) of the reports in the postmarketing safety database that were associated with sildenafil 100 mg were reported as serious events. However, for most of the priapism cases, the reporter indicated that the case was not clinically severe. Also, for most of the priapism cases, the reporter indicated that the patient had recovered or was recovering without sequelae at the time of the report.

The risk of priapism appears to be increased in certain situations. For example, in cases of sildenafil overdose, priapism was reported at a rate more than twice that of the overall postmarketing safety database. Also, across all sildenafil doses, concomitant medication use was reported in 377 reports of priapism, in 27% (102/377) of which the concomitant medication(s) could have contributed to the priapism [i.e. other ED medications, alpha-adrenergic antagonists (phentolamine), psychotropics (amitriptyline, nortriptyline, trazodone, fluphenazine), amphetamines and cocaine]. In the subgroup of cases that reported concomitant use of another ED medication, alprostadil was used concomitantly with sildenafil in 74% (14/19) of the patients of priapism.

As priapism, although rare, is a potentially serious adverse event, the Viagra summary of product characteristics (SmPC) recommends use with caution in patients with anatomic deformation of the penis or in patients who have conditions that may predispose them to priapism (such as sickle cell anaemia, multiple myeloma or leukaemia) (15). Interestingly, sildenafil has been reported to be successful in relieving priapism in patients with sickle cell disease (58).

### NAION

Events suggestive of NAION were not discovered in the DBPC database. In the postmarketing safety database, the reporting rate for events suggestive of NAION was 0.8% (333/39,277). Nearly half (48%) of the cases for which medical history or concomitant medication information was available reported possible predisposing risk factors, including previous ocular disorders (*n* = 52), hypertension (*n* = 58), hyperlipidaemia (*n* = 47), diabetes mellitus and related conditions (*n* = 29), smoking (*n* = 36) and coronary artery disease (*n* = 21). More than half of the events suggestive of NAION were reported by attorneys rather than healthcare professionals and lack appropriate medical information.

No cases of NAION were discovered in a retrospective review of > 13,000 patients in the manufacturer’s clinical trials database or during 2935 patient-years of follow up in a prospective observational study of 3813 men (mean age, 57 years) (59). In another prospective observational study, one case of NAION was identified in a total of 35,569 patient-years of observation, representing an unadjusted NAION incidence estimated to be 2.8 patients per 100,000 patient-years of exposure to sildenafil, which is similar to or lower than estimates in general population samples (59).

The data cited herein do not suggest an increased incidence of NAION in men who took sildenafil for their ED. However, because a causal association between visual field defects and NAION has not been excluded, sildenafil is contraindicated in patients who have loss of vision in one eye because of NAION, regardless of whether this episode was related to previous exposure to a PDE5 inhibitor (15). Should a sudden visual defect occur, the patient should stop taking sildenafil and consult a physician immediately (15).

### Hearing loss

In the DBPC database, events of hearing loss were limited to one case of severe unilateral deafness which is considered to be embolic in aetiology and unrelated to sildenafil treatment and a case of mild deafness that was related to sildenafil treatment. In the latter case, the event is reported as ‘exacerbation of hearing loss,’ implying a relevant medical history, and the patient had concurrent multiple sclerosis.

In the postmarketing safety database, reports were extremely rare, with a reporting rate of 0.01% (3/39,277) for sudden hearing loss and 0.07% (26/39,277) for impaired hearing. The one literature case report in a patient with ED indicated emergence of the event shortly after dosing. This report appears to be isolated, and critical information around dosing and patient characteristics is omitted (60).

Hearing loss is a prevalent condition in the general population and is associated with a number of underlying risk factors, including several drugs. The pharmacological action of sildenafil is not consistent with known ototoxic mechanisms, and there have been no cases that demonstrate the absence of known risk factors and clear evidence of sildenafil challenge/dechallenge or dosing in relation to onset.

### Impaired renal function and hepatic function

Medical history is not a search function that is coded in the postmarketing safety database, but the DBPC database was searched for safety data in men with ED and moderate impairment of renal or hepatic function.

Sildenafil has a low renal clearance (< 2%) and excretion because of high tubular reabsorption in the kidney. Sildenafil is excreted as metabolites predominantly in the faeces (approximately 80% of administered oral dose) and to a lesser extent in the urine (approximately 13% of administered oral dose) (61). In the DBPC database, the sildenafil safety profile in the 21 men with moderate impairment of renal function was similar to that in men with ED and no impairment of renal function. None had a worsening of blood urea nitrogen/urea or creatinine values, and only 2 of 7 (29%) randomised to sildenafil and 9 of 14 (64%) randomised to placebo had adverse events. The adverse events in the two sildenafil recipients (moderate abdominal pain and sciatica; mild peripheral oedema) were not related to sildenafil treatment. Furthermore, published literature suggests that sildenafil is well tolerated in men with ED and receiving dialysis (62), chronic dialysis (63), haemodialysis (64) and peritoneal dialysis (65); across these published trials, in which a total of 86 men received sildenafil, the most common adverse events were similar in frequency, nature and severity to those observed in patients without renal impairment (i.e. headache, flushing, visual disturbances, hypertension, nasal congestion and dyspepsia). Thus, results from approximately 100 men treated with sildenafil in the DBPC database and the published literature suggest that sildenafil is well tolerated in patients with moderate renal impairment. As sildenafil clearance is reduced in patients with severe renal impairment (creatinine clearance < 30 ml/min), a 25-mg initial dose should be considered; based on efficacy and toleration, the dose may be increased to 50 or 100 mg (15).

Sildenafil is extensively and rapidly metabolised by the liver, primarily by CYP3A4 enzymes (15). In men with moderate impairment of hepatic function, the DBPC database suggested a sildenafil safety profile that was similar to that in men with ED and no impairment of hepatic function. Of the 26 men in the DBPC database who had moderate hepatic impairment and were treated with sildenafil, 23% (6/25) experienced a worsening of their alkaline phosphatase, AST, ALT or total bilirubin values, none of which was attributed to sildenafil. Adverse events were experienced by 77% (20/26) of the sildenafil recipients with hepatic impairment compared with 37% (7/19) of the placebo patients with hepatic impairment. In these men, all of the adverse events that were attributed to sildenafil treatment were mild in severity. As sildenafil clearance is reduced in men with hepatic impairment (e.g. cirrhosis), a 25-mg initial dose should be considered; based on efficacy and toleration, the dose may be increased to 50 or 100 mg (15). The safety of sildenafil has not been studied in men with severe hepatic impairment, and its use is therefore contraindicated (15).

### Drug interactions

As sildenafil potentiates the hypotensive effects of nitrates, its concomitant administration with nitric oxide donors (such as amyl nitrite) or nitrates in any form is contraindicated (15). Drug interaction studies have shown that concomitant administration of sildenafil and nitrates is associated with known sildenafil adverse drug reactions (mainly class effects associated with PDE5 inhibition) and consistent reductions in blood pressure (18). Very few men in the DBPC database took nitrates concomitantly with sildenafil (16/8691) or placebo (9/6602), and none of these reported hypotension as an adverse event. Treatment-related adverse events were limited to one man treated with 50 mg who had dyspepsia and one man treated with 100 mg who had periorbital swelling, severe headache and facial flushing. In the postmarketing safety database, concomitant nitrate use represented approximately 1% (478/39,277) of patients, and the most frequent associated adverse events were consistent with the underlying cardiovascular conditions treated by nitrates [e.g. myocardial infarction (18%) and chest pain (15%)] or were hypotensive events from the known pharmacodynamic interaction of sildenafil and nitrates (11%).

Sildenafil metabolism is principally mediated by the CYP450 isoforms 3A4 (major route) and 2C9 (minor route); sildenafil clearance is reduced when administered concomitantly with CYP3A4 inhibitors such as ketoconazole, erythromycin and cimetidine. Concomitant administration of a single 100 mg dose of sildenafil with ritonavir (an HIV protease inhibitor that is a highly potent P450 inhibitor) at steady state (500 mg twice daily) resulted in increases of 300% (4-fold) in the sildenafil maximum serum concentration and 1000% (11-fold) in the sildenafil area under the plasma concentration vs. time curve (15). In the DBPC database, a small number of men took a CYP3A4 inhibitor, most commonly erythromycin or cimetidine, concomitantly with sildenafil (67/8691) or placebo (43/6602). In this subgroup, treatment-related adverse events occurred in nine men taking sildenafil 50 mg and in 11 men taking sildenafil 100 mg, including one man who had treatment-related adverse events at each dose. Treatment-related adverse events were generally known sildenafil adverse drug reactions that occurred at a slightly higher incidence than in the placebo group or in the entire DBPC database. There were too few postmarketing cases (*n* = 19) of concomitant CYP3A4 inhibitor and sildenafil use to make an assessment. Although the Viagra SmPC advises against the concomitant administration of sildenafil with ritonavir and recommends considering a sildenafil starting dose of 25 mg when administered concomitantly with other CYP3A4 inhibitors (15), this collated data review suggests little difference in the safety profile of sildenafil across doses (25–100 mg) when administered concomitantly with CYP3A4 inhibitors.

The current Viagra SmPC advises that patients should be haemodynamically stable on α-blocker therapy before initiating sildenafil treatment, and that initiation of sildenafil at lower doses (25 mg) should be considered (to minimise the potential for developing postural hypotension) (15). The safety of sildenafil administered concomitantly with α-blockers was investigated in three randomised, double-blind, placebo-controlled, cross-over drug interaction trials summarised in the SmPC, in which administration of sildenafil to men with benign prostatic hyperplasia and stabilised on the non-selective α-blocker doxazosin (4 and 8 mg) was associated with mean additional supine blood pressure reductions of 7/7 mmHg (25 mg dose), 9/5 mmHg (50 mg dose) and 8/4 mmHg (100 mg dose) respectively, and with mean additional standing blood pressure reductions of 6/6 mmHg (25 mg dose), 11/4 mmHg (50 mg dose) and 4/5 mmHg (100 mg dose) respectively, but with infrequent reports of symptomatic postural hypotension, which included dizziness and light-headedness, but not syncope) (15). These trials assessed men aged 35–75 years who had documented benign prostatic hyperplasia, had been receiving doxazosin for at least 2 months and took sildenafil after they had taken doxazosin 4 mg once daily for 2 weeks (66). In the first trial, one of four men who took sildenafil 100 mg had a serious adverse event of postural hypotension that began 35 min postdose and lasted for 8 h, but none of the 17 men who took sildenafil 25 mg experienced symptomatic postural hypotension. In the second trial, one of 20 men discontinued prematurely because of hypotension after taking sildenafil 50 mg, but he was also taking minoxidil concomitantly; two other men experienced hypotension as a moderately severe adverse event, with onset 1 h after taking sildenafil 50 mg and resolution of hypotension after 7.5 h. In the third trial, one of 25 men screened with open-label sildenafil 50 mg was discontinued because of symptomatic hypotension (a moderately severe adverse event) 30 min postdose, but none of the 20 men who took sildenafil 100 mg during the trial had a severe adverse event related to blood pressure. In addition to these single-dose, drug interaction trials, several published reports of data from randomised, clinical trials have assessed the safety of sildenafil administered concomitantly with α-blocker therapy and found no evidence of symptomatic postural hypotension associated with concomitant tamsulosin (*n* = 93) or terazosin (*n* = 78) therapy (67), doxazosin therapy (*n* = 14) (68) or alfuzosin therapy (*n* = 21) (69).

In the DBPC database, concomitant α-blocker and study drug administration was uncommon, occurring in only 4.2% (368/8691) of sildenafil recipients and 5.0% (329/6602) of placebo patients. It was also infrequent in the postmarketing safety database, occurring in 4.1% (1600/39,277) of patients. In both databases, non-selective α-blocker use (e.g. doxazosin or terazosin) was reported in most cases. In the DBPC database, the most commonly reported adverse events in sildenafil recipients administered α-blockers concomitantly were dyspepsia, headache and flushing, which are known adverse drug reactions with sildenafil treatment. The incidence of events expected from a pharmacological interaction between sildenafil and an α-blocker (decreased blood pressure, orthostatic hypotension) was very low, and there were no cases of hypotension. Adverse events were similar between men using sildenafil 50 and 100 mg, except for a greater incidence of flushing, dyspepsia and nasopharyngitis in the latter. Of sildenafil recipients with adverse events, 46% (168/349) had treatment-related adverse events, mostly mild events [80% (134/168) mild; 1.8% (3/168) severe]. In comparison, 16.9% (32/189) of placebo patients who were taking an α-blocker had an adverse event that was treatment related, of which 9.4% (3/32) were severe. Overall, sildenafil appears to be well tolerated by men receiving concomitant α-blockers. This collated data review suggests little difference in the safety profile of sildenafil across doses (25–100 mg) with concomitant use of α-blockers.

Guidelines on the treatment of ED recommend PDE5 inhibitors as a first-line treatment (39,70). Other treatments include apomorphine sulphate, vacuum devices, intracavernosal injection with prostaglandin E1 and intraurethral delivery of prostaglandin E1. The safety profile of sildenafil administered concomitantly with other ED treatments has not been investigated in clinical trials and, in the DBPC database, few men used other ED medications concomitantly with sildenafil (13/8691) or placebo (12/6602). Treatment-related events, all of which resolved, occurred in three of these men: mild dyspepsia (alprostadil concomitantly with sildenafil 25 mg), mild chromatopsia and headache (alprostadil concomitantly with sildenafil 50 mg and then 100 mg) and moderate atypical chest pain (sildenafil concomitantly with sildenafil 100 mg). In the postmarketing safety database, 1.7% (670/39,277) of all patients reported concomitant ED medications; approved PDE5 inhibitors in more than half and alprostadil in one-third. In the subgroup that reported concomitant ED medications, the adverse events that occurred at more than twice the rate found in the overall postmarketing safety database usually represented the known dose-related increase in visual effects associated with PDE5 inhibition (cyanopsia and visual disturbance) or a synergistic pharmacological effect on the penis (erection increased, penis disorder and priapism). In most (22/40; 55%) of the patients with visual effects, another PDE5 inhibitor was used concomitantly, and in most (14/19; 74%) of the patients with priapism, alprostadil was used concomitantly. Other events that occurred at more than twice the rate found in the overall postmarketing safety database were related to underlying medical conditions (e.g. pain, dyspnoea and increased blood cholesterol and blood pressure) or to alprostadil injection (e.g. pain). Although the concomitant use of sildenafil with other treatments for ED is advised against in the Viagra SmPC (15), this collated data review identified no unexpected adverse drug reactions, suggesting that the safety risk is likely to be low in the general ED population.

### Overdose

Overdose results from intentionally or inadvertently exceeding the maximum recommended daily dose or dosing frequency of a drug, which for sildenafil is a daily dose of 100 mg and (even if the total daily dose does not exceed 100 mg) a dosing frequency of not more than once per day. Sildenafil has low general toxicity; no relevant reproductive toxicity, genotoxicity or carcinogenic properties; and a substantial safety margin (18,71). Clinical studies of doses higher than the approved maximum showed an increased frequency and severity of known adverse drug reactions, including a dose-related increase in the frequency of visual adverse events, but no clear relationship between dose and maximum decreases in blood pressure and no clinically significant changes in electrocardiograms (18). With administration more frequently than once a day, there was an increased frequency of muscular ache, which was transient and without evidence of muscular damage, and dyspepsia (15,18). In the DBPC database, there were no reports of sildenafil overdose. In the postmarketing safety database, excluding cases in conjunction with suicides or suicide attempts, the reporting rate for sildenafil overdose was 2.3% (884/39,203).

Adverse events in the postmarketing cases of overdose were generally known sildenafil adverse drug reactions (i.e. headache and flushing), which were generally reported at a slightly higher frequency than in the overall postmarketing safety database. Priapism was reported at a rate more than twice that of the overall postmarketing safety database. Of the 884 patients with overdose identified in the postmarketing safety database, 165 reported a first total daily dose > 100 mg. In this subset, the reporting rate was more than twice that for the overall postmarketing safety database for acute myocardial infarction (3.0% vs. 0.7%), myocardial infarction (6.1% vs. 2.5%), tachycardia (2.4% vs. 1.0%), drug interaction (4.8% vs. 2.4%), malaise (2.4% vs. 1.0%), dyspnoea (2.4% vs. 1.0%) and hypotension (5.5% vs. 1.1%). Although a relationship with sildenafil overdose cannot be excluded in 4 of the 12 patients with myocardial infarction and in 14 of the 57 deaths, neither can a relationship between overdose and increased cardiac risk be assumed based on the small number of postmarketing cases. However, we recommend admission to hospital and cardiac monitoring, preferably in a coronary care unit, for 48 h. In this way, any cardiovascular complication can be addressed by experienced healthcare professionals. Standard supportive measures should be adopted as required, but renal dialysis is not expected to accelerate clearance because sildenafil is highly bound to plasma proteins and is not eliminated in the urine.

The highest first total daily dose reported was 2400 mg in a 33-year-old man who took 24 tablets of sildenafil 100 mg. He was diagnosed with annular scotoma, defective colour vision, vascular retinal dilatation, visual field defect and papilloedema. He recovered from all events except for visual field defect and annular scotoma.

Thus, the collated data review determined that overdose with sildenafil is rare in the ED population and identified no new safety issues or adverse reactions in conjunction with overdose. It is noteworthy that, in a parallel-group, double-blind, randomised trial and its long-term open-label extension study, the safety profile of sildenafil, in doses up to 240 mg/day for the treatment of pulmonary arterial hypertension, was unchanged compared with lower dosages (72). In single-dose volunteer studies of doses up to 800 mg, adverse reactions were similar to those seen at lower doses, but the incidence rates and severities were increased (15).

Extensive data (clinical trial data from > 13,000 patients (50,51), 7 years of international postmarketing data, observational studies of > 28,000 men in the United Kingdom (24) and 3813 men in the European Union (73)) was used to support the previously published conclusion that there are no special cardiovascular concerns when sildenafil is used in accordance with product labelling (27).

### Abuse

Abuse can be classified as addiction/dependence (an involuntary compulsion, which is usually marked by tolerance to the effects and withdrawal symptoms when use is terminated), abuse (recreational use without medical rationale but without addiction or dependence) and misuse (intentional or unintentional incorrect use by a healthcare provider or patient). Unintentional misuse includes accidental exposure, drug administration error, incorrect dose administered and medication error. Events relating to addiction/dependence, abuse or misuse of sildenafil were not identified in the DBPC database, but were identified in the postmarketing safety database.

The mode of action of sildenafil is peripheral rather than central (71,74). Consequently, sildenafil lacks the libido-stimulating activity that classically defines an aphrodisiac agent. In addition, there is no evidence that men with ED develop physical dependence or tolerance to sildenafil. The absence of evidence of tolerance developing to the erectogenic effects of sildenafil is further supported by the results of a long-term study over 4 years (75). However, in the postmarketing safety database, sildenafil dependence was reported in a few [0.15% (58/39,277)] patients, mostly psychological dependence on sildenafil to achieve erection or insecurity in initiating intercourse without sildenafil.

Sildenafil abuse represented 0.11% (42/39,277) of all reported events, in 76% of which the patient did not have a diagnosis of ED. Intentional misuse was identified in 1.4% (535/39,277) of all patients, most commonly taking sildenafil for recreational purposes, without a prescription, and/or without a diagnosis of ED (*n* = 207) and adjusting the dose or route/form of administration without direction of a physician (*n* = 141). Unintentional misuse was identified in 0.91% (357/39,277) of patients, including patients who accidentally took the drug, accidentally took more of the drug than intended or altered their dose or dosing frequency without first consulting a physician.

Thus, in the postmarketing safety database, the predominant form of improper use was obtaining sildenafil without a prescription and/or taking sildenafil without a diagnosis of ED. Sildenafil is often obtained through uncontrolled channels outside of the healthcare system, a risky behaviour that is a medical concern that needs to be addressed (76–78). Also, some men take sildenafil for what they perceive as a better sexual performance, even though they do not have ED. No new safety signals or emerging trends were identified from this review of cases reporting sildenafil dependence, abuse and misuse.

## Conclusions

This collated data review found that sildenafil was well tolerated at a dose of 50 or 100 mg, overall, in men who were aged ≥ 65 years and in those who were aged ≥ 75 years. No causal link was identified between sildenafil administered in accordance with product labelling and cardiovascular events, and further support was lent to the safety of sildenafil in men at risk for cardiovascular disease, in that no new safety risks relating to cardiovascular events were identified in patients with cardiovascular conditions, hypertension or diabetes mellitus. No new safety risks were identified with sildenafil relating to priapism or NAION. In the small number of men with ED and moderate impairment of renal function or hepatic function who have been treated with sildenafil in DBPC trials, the sildenafil safety profile was similar to that in men with ED and no impairment of renal or hepatic function. As sildenafil was well tolerated, has a relatively short half-life (4–5 h) and is administered as needed in a maximum of one dose per day, the safety risk from drug interactions is likely to be low in the general ED population. Indeed, no unexpected adverse drug reactions were identified from the concomitant use of sildenafil with nitrates, nitric oxide donors, CYP3A4 inhibitors, other ED medications or α-blockers. Overdose with sildenafil was rare in the ED population, and no new adverse reactions were identified in conjunction with overdose, dependence, abuse or misuse. It can be concluded that sildenafil in all registered doses has a good safety profile.

## Footnotes

1 PubMed search: ‘erectile dysfunction’ (Mesh) and ‘drug toxicity’ (Mesh) and (sildenafil or vardenafil or tadalafil) and [‘age factors’ (Mesh) or ‘age groups’ (Mesh)].

2 The recommended starting dose is 50 mg taken as needed approximately 1 h before sexual activity [VIAGRA® (sildenafil citrate) summary of product characteristics, http://www.emea.europa.eu/humandocs/PDFs/EPAR/viagra/H-202-PI-en.pdf]. Based on efficacy and toleration, the dose may be increased to 100 mg or decreased to 25 mg. The maximum recommended dose is 100 mg. The maximum recommended dosing frequency is once per day.
